# Variation in survival of women with breast cancer: Health Board remains a factor at 10 years

**DOI:** 10.1054/bjoc.2001.1957

**Published:** 2001-09

**Authors:** C J Twelves, C S Thomson, J A Dewar, D H Brewster, on behalf of the Scottish Cancer Therapy Network

**Affiliations:** Cancer Research Campaign Department of Medical Oncology, Beatson Oncology Centre, Glasgow, G11 6NT; Scottish Cancer Intelligence Unit, Information and Statistics Division, Trinity Park House, Edinburgh, EH5 3SQ; Department of Radiotherapy and Oncology, Ninewells Hospital and Medical School, Dundee, DD1 9SY, UK

**Keywords:** breast cancer, survival, regional variations in survival

## Abstract

Multivariate survival analysis of women with breast cancer diagnosed in 1987 with at least 10 years follow-up confirmed variations depending on the Health Board of their treatment. Differences in survival according to surgical caseload/specialization or deprivation were not statistically significant. © 2001 Cancer Research Campaign http://www.bjcancer.com


*British Journal of Cancer* (2001) **85**(5), 637–640
© 2001 Cancer Research Campaign
doi: 10.1054/ bjoc.2001.1957, available online at http://www.idealibrary.com on IDEAL

http://www.bjcancer.com

### Variation in survival of women with breast cancer: Health Board remains a factor at 10 years

**CJ Twelves[1], CS Thomson[2], JA Dewar[3] and DH Brewster[2] on behalf of the Scottish Cancer Therapy Network**

[1]Cancer Research Campaign Department of Medical Oncology, Beatson Oncology Centre, Glasgow G11 6NT; [2]Scottish Cancer Intelligence Unit, Information and Statistics Division, Trinity Park House, Edinburgh EH5 3SQ; and [3]Department of Radiotherapy and Oncology, Ninewells Hospital and Medical School, Dundee DD1 9SY, UK

**Summary** Multivariate survival analysis of women with breast cancer diagnosed in 1987 with at least 10 years follow-up confirmed variations depending on the Health Board of their treatment. Differences in survival according to surgical caseload/specialization or deprivation were not statistically significant. © 2001 Cancer Research Campaign http://www.bjcancer.com

**Keywords**: breast cancer; survival; regional variations in survival

We previously presented 5-year survival analyses for all women in Scotland diagnosed with breast cancer during 1987 (Twelves et al, 1998). That study demonstrated geographical variation in survival that persisted even after adjustment for known prognostic factors. This variability in survival appeared to be partly related to selection for surgery but also to differences in systemic adjuvant treatment. In contrast to earlier findings, after adjustment for clinical factors our data did not confirm a significant effect of surgical specialization (Gillis and Hole, 1996), caseload (Sainsbury et al, 1995) or deprivation (Schrijvers et al, 1995; Karjalainen and Pukkala, 1990) on survival. Here, we investigate whether our findings are confirmed with follow-up beyond 10 years.

### PATIENTS AND METHODS

### Study population

A national, population-based study of all women with invasive breast cancer recorded by the Scottish Cancer Registry was undertaken by the Scottish Cancer Therapy Network (SCTN). The current study reports the 10-year survival of women diagnosed with breast cancer in 1987 who had no evidence of metastases at presentation and underwent surgery as part of their primary treatment.

### Data collection and analysis

All case notes were reviewed and information on referral patterns, clinical characteristics, tumour pathology and primary treatment extracted. Demographic data included age at diagnosis. Health Board (HB; equivalent to the Health Authorities elsewhere in the UK) of first treatment and the Carstairs deprivation index (Carstairs and Morris, 1991). A further probability linkage (Kendrick and Clarke, 1993) to the Registrar General's death records to the end of 1998 provided updated survival data. The endpoint was death from any cause. Prior to undertaking these analyses, a review of the database revealed small numerical differences. These do not substantially affect our main conclusions at 5 years, although the higher hazard ratio of death in Fife HB compared with Greater Glasgow Health Board (GGHB) is no longer significant.

Kaplan-Meier univariate survival analyses were performed on all clinical, treatment and service-related factors. Cox's proportional hazards multivariate analysis was then undertaken in two models. One included clinical factors plus significant service-related factors; the other included clinical factors and significant treatment factors.

### RESULTS

In all, 2581 patients were recorded with breast cancer in 1987; of these, 135 were ineligible for analysis, most often because they had in situ disease only. Case notes could not be located for a further 252 patients and 79 cases were death certificate only registrations, leaving 2115 (87%) potentially eligible patients available for analysis. Another 175 patients with metastatic disease at presentation and the 323 women who did not undergo definitive surgery were also excluded, so 1617 women were included in this study population.

The overall Kaplan-Meier estimates of survival at 5 and 10 years for women with breast cancer diagnosed in 1987 were 70.9% (95% CI: 68.7%, 73.1%) and 52.7% (50.3%, 55.1%), respectively. In the univariate analyses all clinical factors (age, clinical stage, oestrogen receptor status, pathological node status and pathological tumour size), service-related factors (HB, surgical caseload and deprivation) and treatment factors (type of surgery and use of adjuvant chemotherapy) that had been significant at 5 years remained significant at 10 years (data not shown).

In the multivariate analysis (Table 1), the clinical factors retained significance (all $P < 0.001$). By contrast, treatment factors were not significant at either 5 or 10 years. Amongst the service-related factors, there was a trend for deprivation and surgical caseload to be associated with poorer survival, but these remained non-significant in the multivariate model with follow-up beyond 10 years. There was, however, significant variation in the risk of

*Received 5 February 2001*
*Revised 10 May 2001*
*Accepted 1 June 2001*

*Correspondence to:* CJ Twelves

BJOC 01-1957 637-640 20/8/01 3:23 pm Page 638

**Table 1** Adjusted hazard ratios of death with a minimum of 6 and 11 years follow-up, from Cox regression

| **Clinical factors** | **Adjusted hazard of death (95% CI) with deaths to end 1993** | ***P* value** | **Adjusted hazard of death (95% CI) with deaths to end 1998** | ***P* value** |
|---|---|---|---|---|
| Age group (years) | | 0.0059[a] | | < 0.0001[a] |
| < 50 | 1.0 | | 1.0 | |
| 50–64 | 1.02 (0.83, 1.27) | | 1.22 (1.01, 1.46) | |
| 65–79 | 1.12 (0.90, 1.40) | | 1.50 (1.24, 1.81) | |
| > = 80 | 1.94 (1.35, 2.79) | | 2.81 (2.07, 3.81) | |
| Clinical stage | | 0.0003[a] | | 0.0003[a] |
| I | 1.0 | | 1.0 | |
| II | 1.45 (1.10, 1.92) | | 1.41 (1.13, 1.76) | |
| III | 2.08 (1.48, 2.92) | | 1.85 (1.40, 2.46) | |
| Not known | 1.58 (1.16, 2.16) | | 1.42 (1.11, 1.83) | |
| Oestrogen receptor status | | < 0.0001[a] | | < 0.0001[a] |
| Positive | 1.0 | | 1.0 | |
| Negative | 2.13 (1.71, 2.66) | | 1.86 (1.55, 2.23) | |
| Not known | 1.42 (1.13, 1.79) | | 1.28 (1.06, 1.54) | |
| Node status by tumour size interaction | | < 0.0001[a] | | < 0.0001[a] |
| Node positive | | | | |
| ≤ 2 cm | 4.70 (2.69, 8.23) | | 3.49 (2.34, 5.21) | |
| > 2 cm | 5.23 (3.05, 8.95) | | 3.88 (2.65, 5.66) | |
| Not known | 5.29 (2.95, 9.46) | | 3.74 (2.44, 5.74) | |
| Node INS[b] | | | | |
| ≤ 2 cm | 1.43 (0.73, 2.79) | | 1.22 (0.76, 1.97) | |
| > 2 cm | 3.12 (1.70, 5.71) | | 2.26 (1.45, 3.53) | |
| Not known | 2.18 (1.03, 4.61) | | 2.02 (1.18, 3.45) | |
| Node negative[c] | | | | |
| ≤ 2 cm | 1.0 | | 1.0 | |
| > 2 cm | 3.29 (1.78, 6.07) | | 2.28 (1.45, 3.57) | |
| Not known | 1.50 (0.65, 3.43) | | 0.91 (0.46, 1.81) | |
| Node not known | | | | |
| ≤ 2 cm | 2.75 (1.55, 4.88) | | 2.21 (1.47, 3.32) | |
| > 2 cm | 4.16 (2.34, 7.40) | | 3.01 (1.99, 4.57) | |
| Not known | 3.77 (2.08, 6.81) | | 3.15 (2.05, 4.83) | |
| **Service factors** | | | | |
| Health Board | 0.04[a] | | 0.0044[a] | |
| Ayrshire and Arran | 1.46 (1.06, 2.01) | | 1.50 (1.15, 1.96) | |
| Borders | 1.24 (0.59, 2.59) | | 1.40 (0.80, 2.47) | |
| Argyll and Clyde | 1.48 (1.05, 2.10) | | 1.43 (1.07, 1.90) | |
| Fife | 1.30 (0.87, 1.93) | | 1.17 (0.84, 1.62) | |
| GGHB | 1.0 | | 1.0 | |
| Highland | 1.03 (0.65, 1.64) | | 0.96 (0.64, 1.42) | |
| Islands[d] | 0.66 (0.32, 1.39) | | 0.77 (0.43, 1.38) | |
| Lanarkshire | 1.19 (0.85, 1.65) | | 1.12 (0.85, 1.47) | |
| Grampian | 0.95 (0.69, 1.32) | | 0.96 (0.73, 1.25) | |
| Lothian | 0.83 (0.61, 1.14) | | 0.79 (0.61, 1.02) | |
| Tayside | 1.34 (0.95, 1.89) | | 1.19 (0.90, 1.59) | |
| Forth Valley | 1.49 (0.95, 2.33) | | 1.31 (0.89, 1.92) | |
| Dumfries and Galloway | 1.09 (0.69, 1.72) | | 0.93 (0.63, 1.38) | |
| Surgical caseload[e] | | 0.61[f] | | 0.18[f] |
| 1–9 cases | 1.07 (0.83, 1.37) | | 1.12 (0.91, 1.38) | |
| 10–29 | 1.11 (0.91, 1.35) | | 1.17 (0.99, 1.38) | |
| Team ⩾ 30 | 1.0 | | 1.0 | |
| Referral to oncologist[g] | | 0.21[f] | | 0.52[f] |
| Never/not primary | 1.0 | | 1.0 | |
| Yes | 1.12 (0.94, 1.34) | | 1.05 (0.91, 1.22) | |
| Deprivation[h] | | 0.28[f] | | 0.30[f] |
| Least deprived | 1.0 | | 1.0 | |
| Intermediate | 1.13 (0.92, 1.38) | | 1.05 (0.89, 1.24) | |
| Most deprived | 1.24 (0.95, 1.61) | | 1.19 (0.95, 1.48) | |
| **Treatment factors** | | | | |
| Type of surgery | | 0.84[f] | | 0.32[f] |
| Mastectomy | 1.0 | | 1.0 | |
| Breast conservation | 0.98 (0.81, 1.19) | | 0.92 (0.78, 1.08) | |
| Adjuvant radiotherapy | | 0.83[f] | | 0.85[f] |
| Given | 1.02 (0.85, 1.22) | | 0.99 (0.85, 1.15) | |
| Not given | 1.0 | | 1.0 | |

 *© 2001 Cancer Research Campaign*

**Table 1** Contiinued

| Treatment factors | Adjusted hazard of death (95% CI) with deaths to end 1993 | *P* value | Adjusted hazard of death (95% CI) with deaths to end 1998 | *P* value |
|---|---|---|---|---|
| Adjuvant chemotherapy | | 0.92[f] | | 0.53[f] |
| Given | 0.98 (0.72, 1.34) | | 1.09 (0.83, 1.42) | |
| Not given | 1.0 | | 1.0 | |
| Adjuvant endocrine therapy | | 0.43[f] | | 0.16[f] |
| Given | 0.93 (0.77, 1.12) | | 0.89 (0.76, 1.05) | |
| Not given | 1.0 | | 1.0 | |
| Adjuvant chemotherapy or endocrine therapy | | 0.28[f] | | 0.15[f] |
| Given | 0.90 (0.74, 1.09) | | 0.89 (0.76, 1.04) | |
| Not given | 1.0 | | 1.0 | |

[a]*P* Values are Wald statistics for entry, adjusted for all other significant factors; [b]INS=inadequate negative sample = 1, 2, 3, unknown number taken, all negative; [c]Negative = 4 or more nodes taken, all negative; [d]Due to the small numbers of cases, the Orkney, Shetland and Western Isles Health Boards were analysed together as a single group, representing off-mainland care; [e]Note that the 12 cases where the surgeon was unknown were not included in the analysis; [f]*P* values are Wald statistics for entry, adjusted for significant clinical factors only; [g]Note that the 29 cases where the date of referral to the oncologist was unknown were not included in the analyses because it was not possible to determine whether or not this was part of primary treatment; [h]Note that a deprivation score could not be assigned for one case.

death across the HBs at 5 years (*P* = 0.04). This effect was stronger at 10 years (*P* = 0.004), with two HBs having a significantly higher risk of death than GGHB. When the multivariate analyses were extended to include survival of all 1940 women with non-metastatic disease (i.e. the 1617 surgical, non-metastatic cases and the 323 non-surgical cases, non-metastatic), at 5 years HB was of borderline non-significance (*P* = 0.055); with 10 years follow-up became statistically significant (*P* = 0.004) in this broader group. Likewise, HB was also significant in a multivariate model limited to breast cancer specific deaths (*P* = 0.003).

### DISCUSSION

This study shows that the effect of the clinical factors on survival remained significant with at least 10 years follow-up. More importantly, the proposition that HB affected survival at 5 years was strengthened with longer follow-up. However, treatment factors remained non-significant in the multivariate model, reflecting the determination of treatment by clinical factors (e.g., patients with poorer prognoses are more likely to receive chemotherapy).

Previously, we were unable to demonstrate the beneficial effect on survival of being managed by a higher caseload or specialist surgeon (Sainsbury et al, 1995; Gillis and Hole, 1996) nor by being resident in affluent areas (Schrijvers et al, 1995; Karjalainen and Pukkala, 1990). Even with substantially more events (842 compared with 584) we still cannot substantiate those findings. However, this study probably has insufficient power to exclude a potentially important effect of deprivation (Thomson et al, 2001) or case load on survival.

The effect of HB on survival was of borderline significance at 5 years, but at 10 years it is clear and statistically significant (*P* = 0.004). In the multivariate models, adjusted survival across HBs ranged from 67% to 84% at 5 years and 43% to 65% at 10 years. The reason for this effect of HB remains unclear. It is not easily attributed to differences in the distributions of clinical factors across the HBs because these have been incorporated in the multivariate model. Similarly, differences in survival persist when only breast cancer specific deaths were considered, suggesting that the risk of a non-breast cancer death was similar across the HBs. The proportions of women who had non-metastatic disease, but did not undergo definitive surgery, varied widely between HBs (range 6.2–31.0%). With 5-year follow-up there appeared to be selection bias due to surgery since HB was an independent predictor of outcome only if the analysis was limited to surgical patients. However, at 10 years this bias was not seen and HB had an independent effect on survival in all non-metastatic patients irrespective of whether they underwent definitive surgery.

The apparently strengthened effect of HB on survival is, however, compatible with geographical differences in the use of adjuvant systemic therapy as the Early Breast Cancer Trialists' Collaborative Group (EBCTCG) overview showed that survival curves typically diverge with time in adjuvant trials (EBCTCG, 1992). Although adjuvant systemic treatment appeared not to influence outcome in the multivariate model, this can be explained by the nature of the disease determining such treatment. Moreover, the overview hypothecated a 6% survival benefit at 10 years (EBCTCG, 1992) and, despite the additional events, the current study still has only approximately 35% power to detect an effect of this magnitude.

Further follow-up confirms that, historically at least, the HB of their treatment was an important determinant of survival in women with breast cancer. The most likely explanation is differences in adjuvant systemic (EBCTCG, 1992) or local (EBCTCG, 2000) treatment between HBs. Indeed, this may explain the apparent effect of surgical caseload and specialization previously described (Sainsbury et al, 1995; Gillis and Hole, 1996). Interest now lies in whether this effect of HB remains in women diagnosed more recently, when the use of adjuvant systemic treatment appeared uniform across the HBs (Scottish Breast Cancer Focus Group, 1996).

### FUNDING

The Scottish Cancer Therapy Network is funded by grants from the Clinical Resource and Audit Group and the Chief Scientist Office, both of the Scottish Executive Health Department. The views expressed are, however, those of the authors.

### ACKNOWLEDGEMENTS

The authors would like to thank the SCTN data managers for their help with data validation and support, and Diane Stockton for her comments on the text.

*© 2001 Cancer Research Campaign*

### REFERENCES

Carstairs V and Morris R (1991) *Deprivation and Health in Scotland*. Aberdeen University Press: Aberdeen

Early Breast Cancer Trialists' Collaborative Group (1992) Systemic treatment of early breast cancer by hormonal, cytotoxic or immune therapy. *Lancet* **339**: 1–15, 71–85

Early Breast Cancer Trialists' Collaborative Group (2000) Favourable and unfavourable effects on long-term survival of radiotherapy for early breast cancer: an overview of the randomised trials. *Lancet* **355**: 1757–1770

Gillis CR and Hole DJ (1996) Survival outcome of care by specialist surgeons in breast cancer: a study of 3786 patients in the West of Scotland. *BMJ* **312**: 145–148

Karjalainen S and Pukkala E (1990) Social class as a prognostic factor in breast cancer survival. *Cancer* **66**: 819–826

Kendrick S and Clarke J (1993) The Scottish Record Linkage System. *Health Bulletin* **51**: 72–79

Sainsbury R, Haward B, Rider L et al (1995) Influence of clinician workload and patterns of treatment on survival from breast cancer. *Lancet* **345**: 1265–1270

Schrijvers CTM, Mackenbach JP, Lutz JM et al (1995) Deprivation and survival from breast cancer. *Br J Cancer* **72**: 738–743

Scottish Breast Cancer Focus Group, Scottish Cancer Trials Breast Group, Scottish Cancer Therapy Network (1996) *Scottish Breast Cancer Audit 1987 and 1993*. Scottish Cancer Therapy Network: Edinburgh

Thomson CS, Hole DJ, Twelves C, Brewster DH and Black RJ (2001) Prognostic factors in women with breast cancer: distribution by socioeconomic status and effect on differences in survival. *J Epidemiol Community Health* **55**: 308–315

Twelves CJ, Thomson CS, Gould A et al (1998) Variation in the survival of women with breast cancer in Scotland. *Br J Cancer* **78**: 566–571

 *© 2001 Cancer Research Campaign*
